# Recent Advances in Biliopancreatic Endoscopy

**DOI:** 10.3390/medicina58050593

**Published:** 2022-04-26

**Authors:** Andrea Anderloni, Kareem Khalaf

**Affiliations:** 1 Gastroenterology and Digestive Endoscopy Unit, Fondazione I.R.C.C.S. Policlinico San Matteo, Viale Camillo Golgi, 19, 27100 Pavia, Italy; 2 Digestive Endoscopy Unit, Department of Gastroenterology, Humanitas Research Hospital-IRCCS, 20089 Rozzano, Italy; kareem.khalaf@st.hunimed.eu

In this Special Issue of *Medicina* entitled “Recent Advances in Biliopancreatic Endoscopy” in the Section “Gastroenterology & Hepatology”, nine articles pave new insights into the advances in the world of biliopancreatic endoscopy. This Special Issue deals with a wide range of endoscopic diagnostic and therapeutic techniques. Since its inception in the 1980s, endoscopic ultrasound (EUS) technology has progressed substantially, moving from a supplementary add-on to a key modality in the diagnosis and treatment of a wide range of disorders. EUS initially provided clinicians with valuable clinical and anatomic information focused on various gastrointestinal diseases. Aspects of a structure’s shape, size, and location reveal a diverse range of descriptive variables in a variety of cases. The first article reported a research study on the clinical yield of string sign in endoscopic ultrasound (EUS) whilst diagnosing pancreatic cysts [1]. Interestingly, a higher level of accuracy in predicting mucinous cystic neoplasms provides an insight into the clinical diagnostic ability of encompassing fluid string sign as a factor in our pursuit of diagnosing mucinous neoplasms. As a preferred method of radiological options, EUS predilection arises from the ability to acquire fluid samples. In this Special Issue, we turn our focus to an article that reviewed the role of contrast-enhanced EUS (CE-EUS) in therapeutic interventions. Strikingly, EUS-guided procedures under contrast enhancement provide a parallel to high efficacy in many interventions [2]. Primarily, tissue acquisition and tumor ablation under CE-EUS facilitate novel management options that previously required percutaneous or invasive surgical approaches to an endoscopic alternative with higher efficacious success rates and less adverse events. Another article in this Special Issue reviewed the combination of EUS with complex high competence techniques such as Endoscopic Retrograde Cholangiopancreatography (ERCP) [3]. Since the 1960s, ERCP has progressed to become not only a vital diagnostic tool but also an effective therapeutic intervention in the treatment of a variety of hepatobiliary diseases. A specialized side-viewing endoscope is guided into the duodenum, allowing devices to access the biliary and pancreatic ducts. The therapy of biliary blockages for benign and malignant causes is a common biliary procedure performed by ERCP. The ability to obtain a full diagnostic representation that is mutually beneficial for attaining technical success and positive clinical outcome in ERCP is regarded as a new ‘endoscopic artform’. Furthermore, as an interesting addendum perspective to this Special Issue, the article entitled “Informed Consent for Endoscopic Biliary Drainage: Time for a New Paradigm” provides an explanation of the importance of obtaining informed consent for EUS prior to any ERCP procedure, in order to limit the exposure of patients to multiple procedures when both can be incorporated into one [4]. Ultimately, the concept here is to obtain consent from patients regarding solutions to a specific problem rather than a specific procedure. Understanding the context and structures of obtaining informed consent enables a positive directive in any therapeutic management of a disease. Next, we turn our focus to a systematic review in this Special Issue. Advances in therapeutic options for altered biliopancreatic anatomy have presented a major problem for many endoscopists [5]. The success rates considered for endoscopic guided therapies may be reduced while aligning with a low adverse event rate with detailed consideration of multiple factors. One solution to this conundrum is a multidisciplinary approach towards such patients for better overall outcomes.

On another note, management techniques reviewed in this Special Issue provide awareness of indications regarding the timing of interventions [6,7]. In the article entitled “Direct Endoscopic Necrosectomy: Timing and Technique”, the authors provide an evaluation of previous studies that encompassed a known complication of pancreatitis, walled-off pancreatic necrosis [8]. Necrotic collections need to be treated promptly and the differentiation under EUS offers an interventional technique that may be integrated in clinical practice [9,10]. Aspects evaluating technical features of the procedure and the different flow and apparatus used deliver a global notion of similarity to critical complications in routine practice. 

The use of lumen-apposing metal stents is an emerging therapy alternative to surgery, offering a minimally invasive option. Tarantino et al. conducted a web-based survey of endoscopists regarding expertise, peri- and intra-procedural aspects of EUS gastroenteric anastomosis (EUS-GEA) performance [11]. Of the sixty endosonographers asked to participate, 89.7% reported that EUS-GEA would be beneficial in their daily clinical practice. Although 66.6% reported the procedure was technically difficult, 82.8% reported the risk of adverse events was acceptable when evaluating the end benefits. There are wide variations in practice and studies, such as those mentioned previously, which provide indications that define technical, qualitative and peri-procedural unity in practice.

Recent studies have explored improvements inendoscopic diagnostic and therapeutic procedures. In this Special Issue of *Medicina*, we extol five articles reviewing techniques on necrosectomy, lithotripsy techniques of biliary stones, risk stratification of EUS and ERCP in the same session, with an additional follow-up perspective commentary that stipulates the use of informed consent prior to ERCP for EUS to decrease additional procedures. We also present an original piece studying the analytic yield of string sign and mucinous pancreatic cystic neoplasms which have enhanced the notion of diagnostic accuracy in a clinical setting. Overall, this Special Issue establishes important ideals in the world of biliopancreatic endoscopy. Regarding the concept of a biliopancreatic approach that focuses more on pathology than procedure, we find ourselves taking the appropriate route to finding answers to many pathologies. We must rid our thought process of the understatement that a procedure is necessarily what a patient needs, but also be malleable in providing a complete biliopancreatic management option in order to correctly manage the patient’s needs.
